# Barriers to preventive therapy for breast and other major cancers and strategies to improve uptake

**DOI:** 10.3332/ecancer.2015.595

**Published:** 2015-11-24

**Authors:** Andrea DeCensi, Mangesh A Thorat, Bernardo Bonanni, Samuel G Smith, Jack Cuzick

**Affiliations:** 1 Division of Medical Oncology, E.O. Ospedali Galliera, Mura delle Cappuccine 14, Genoa 16128, Italy; 2 Centre for Cancer Prevention, Wolfson Institute of Preventive Medicine, Queen Mary University of London, London EC1M 6BQ, UK; 3 Breast Services, Division of Surgery and Interventional Science, Whittington Hospital, Magdala Avenue, London N19 5NF, UK; 4 Division of Cancer Prevention and Genetics, European Institute of Oncology, Via Ripamonti 435, Milan 20141, Italy; 5 Health Behaviour Research Centre, University College London, London WC1E 7HB, UK

**Keywords:** aspirin, biomarkers, breast cancer, cancer, drug repurposing, preventive therapy, SERMs

## Abstract

The global cancer burden continues to rise and the war on cancer can only be won if improvements in treatment go hand in hand with therapeutic cancer prevention. Despite the availability of several efficacious agents, utilisation of preventive therapy has been poor due to various barriers, such as the lack of physician and patient awareness, fear of side effects, and licensing and indemnity issues. In this review, we discuss these barriers in detail and propose strategies to overcome them. These strategies include improving physician awareness and countering prejudices by highlighting the important differences between preventive therapy and cancer treatment. The importance of the agent–biomarker–cohort (ABC) paradigm to improve effectiveness of preventive therapy cannot be overemphasised. Future research to improve therapeutic cancer prevention needs to include improvements in the prediction of benefits and harms, and improvements in the safety profile of existing agents by experimentation with dose. We also highlight the role of drug repurposing for providing new agents as well as to address the current imbalance between therapeutic and preventive research. In order to move the field of therapeutic cancer prevention forwards, engagement with policymakers to correct research imbalance as well as to remove practical obstacles to implementation is also urgently needed.

## Introduction

**Figure 1.**
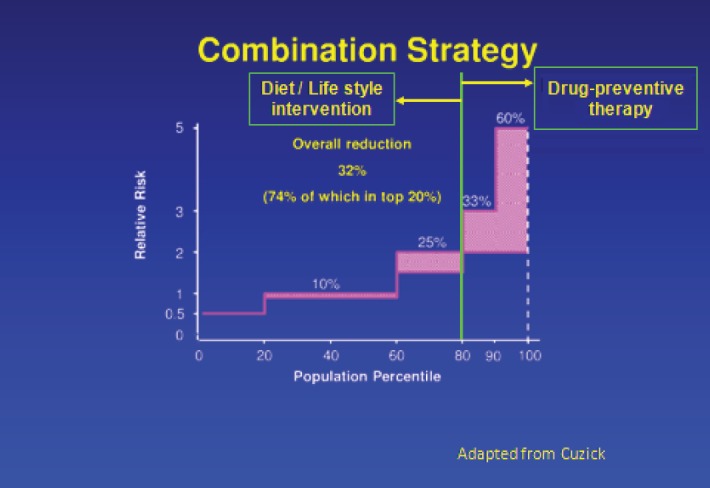
Combination strategy to reduce breast cancer burden worldwide.

Significant improvements in cancer treatments have occurred over the past few decades, but our cancer prevention efforts have not kept pace with them. As a result, the global cancer burden continues to rise. Therapeutic cancer prevention is an effective and essential tool in our fight against cancer, and yet utilisation of preventive therapy has been woefully inadequate. Use of drugs to cut risk by 40–50% in the top 20% of the general population can achieve a 30% reduction in the cancer burden (Figure 1). However, unlike early detection/screening interventions, at an individual level, the success of therapeutic prevention goes unnoticed, while the side effects get noticed, leading to a failure to appreciate its value within the medical profession. To elucidate this point further, an overdiagnosed case that would not become symptomatic within the person’s lifetime is actually a failure of a screening intervention, yet it often gets considered as a success by the public because from this individual’s point of view, his/her life was saved by cancer being caught early. For preventive therapy, we cannot identify individuals whose cancer was prevented or risk was substantially reduced because of the lack of measurable biomarkers of efficacy, which currently exist for other diseases, including cardiovascular diseases, prevention of diabetes complications or osteoporotic bone fractures. Therefore, from that person’s point of view, they either took medication unnecessarily or in the worst case scenario, unnecessarily suffered the adverse effects of such therapy. Thus, preventive therapy for cancer is often discounted as overtreatment and used as an example of overmedicalisation. Understanding and overcoming such perception differences, along with other barriers, is essential if we are to realise the full potential of this approach for cancer control. In this short review, we discuss important barriers to therapeutic cancer prevention, strategies to overcome these barriers and future research needs (Table 1), using breast cancer prevention as the main example.

## Breast cancer prevention

Breast cancer incidence continues to increase with an estimated 1.6 million cases occurring worldwide each year and it is now the commonest cancer in women, making it an ideal cancer for prevention. Strong evidence now exists from nine large phase-III trials that preventive therapy with selective oestrogen receptor modulators (SERMs) reduces breast cancer incidence by 40% [1]. More recently, aromatase inhibitors (AIs) have also shown a more than 50% reduction in breast cancer in two phase-III trials in postmenopausal women [2, 3].

In 1998, the US FDA approved tamoxifen for primary breast chemoprevention in both premenopausal and postmenopausal women at high risk based on the Gail model. In 2007, the food and drug administration (FDA) also approved raloxifene for primary breast cancer chemoprevention for postmenopausal women using the same risk model. Current ASCO guidelines suggest including tamoxifen in discussion of risk reduction strategies for high-risk women [4–6]. The UK National Institute for Health and Care Excellence (NICE) guidelines also recommend tamoxifen or raloxifene as preventive therapy in women with lifetime breast cancer risk above 30% [7].

## Low uptake of preventive therapy for breast cancer

Tamoxifen uptake for eligible high-risk women remains very low primarily due to concerns about side effects and lack of demonstrated mortality reduction [6, 8]. Even for premenopausal women attending high-risk clinics where the risk benefit ratio of tamoxifen is largely favourable, the rate of tamoxifen uptake has been reported to be only 10% [9] and is regarded as a preference-sensitive decision [8–10]. It is estimated that about 7% of 50-year-old women would be eligible for preventive therapy based on the NICE criteria of a >5% 10-year risk, and this number would approach 15% if SNP scores and mammographic density were also used [11–13]. The uptake of breast cancer preventive therapy has been below 1% in spite of a potential population of approximately 10 million high-risk women aged 35–79 years in the United States according to FDA criteria [14] and similar figures in Europe who may be eligible for breast cancer preventive therapy according to high-risk assessment models. In 2010, the vast majority of women reporting use of preventive therapy for breast cancer prevention in the USA were using raloxifene; 96,890 [95% confidence interval (CI), 41,277–192,391] of approximately 45 million women aged 50–79 years were taking raloxifene as compared to 20,598 (95% CI: 518–114,864) of approximately 77 million women aged 35–79 years using tamoxifen [15]. The prevalence of preventive therapy was however very low for both drugs – 0.03% (95% CI: 0.001–0.15) for tamoxifen and 0.21% (95% CI: 0.09–0.43) for raloxifene [15]. Data from the US National Health Interview Surveys show that breast cancer preventive therapy use was similar in years 2000 and 2005, and the overall pictures suggests a shift away from tamoxifen and towards raloxifene for this purpose [16].

## Reasons for the low uptake

Low uptake of preventive therapy is a result of multitudes of factors [17]. An important factor is physicians’ lack of knowledge about preventive therapy. Also important are their prejudices, lack of ability to make accurate risk predictions and communication challenges. It is important to distinguish between oncologists in breast clinics who regularly prescribe drugs such as tamoxifen and AIs for the treatment of cancer and general practitioners (GPs) or family physicians who do not routinely prescribe these drugs. GPs are often the first point of contact for a woman to discuss her concern regarding breast cancer risk and prevention, and women often do not get a chance to discuss this with an oncologist or genetic risk counsellor. Nononcologist physicians may be reluctant to prescribe tamoxifen due to concerns about side effects or their own lack of sufficient information about risk reduction options [18–20]. In a study of physicians in California, Kaplan *et al* reported that frequent barriers to initiating cancer prevention counselling were ‘not enough time’ (40.3%) and ‘insufficiently informed about risk reduction options’ (19.1%) [18]. This is particularly important since a physician’s recommendation is a key factor in a person’s decision [20, 21]. Similarly, data from a clinical trial also suggest that a dedicated caregiver–participant relationship is a critical component for an elevated participation [22]. In an Italian study of over 265 subjects who entered a prevention trial of tamoxifen at low dose and 192 who declined to participate [22], the three most frequent reasons for entry were willingness to participate in a research programme (60%), the need/desire to receive frequent medical care (58%), and the desire to contribute to medical knowledge (44%), whereas the top three reasons for refusal were fear of medication abuse (33%), concern about adverse effects (31%), and advice against enrolment by their family or specialist doctor (24%) [22]. While family history is a common factor in a physician’s consideration of cancer prevention for his/her patient, other risk factors like diagnosis of proliferative or atypical benign breast disease [23] are often overlooked – despite clear evidence of a significantly higher breast cancer risk in such women. These issues underscore the need to establish a personal relationship between the high-risk individual and a well-informed caregiver. However, efforts to increase awareness of preventive therapy among physicians [24–26], including calls for developing support tools [27] have been adversely impacted by scepticism of other opinion leaders [28, 29]. Unsurprisingly, in a recent survey conducted among over 200 breast cancer specialists during different breast cancer meetings in Italy and Switzerland or sent electronically among the alumni of the European School of Oncology, the top three important or very important reasons for low uptake were the following: (1) drugs may have serious side effects; (2) no evidence for a reduction in mortality; (3) drugs are off label in Europe (manuscript in preparation). The issue of lack of effect on mortality discussed in detail below is a main point of contention. This suggests that the differences between use of drugs for cancer treatment and cancer prevention, and the need of a different approach/thinking for each are not always sufficiently appreciated by clinicians regularly treating cancer. Surmounting this obstacle may require a cultural change in the oncology community.

A high-risk individual’s personal reluctance to undergo preventive therapy is determined by several factors. These include underestimation of benefits and/or overestimation of harms [20, 30]. Our inability to accurately estimate risk in certain high-risk categories like benign breast diseases further aggravates this problem [16, 23] and research to improve our risk-prediction models urgently needed [23]. However, reluctance has been reported even in those situations where individuals clearly recognise [31] a net benefit with preventive therapy [20, 30] and provision of information [32] through educational interventions [8] does not improve uptake. The fear of side effects was the main reason for not electing to take preventive therapy in a study by Port *et al* [30]. Behavioural research indicates that a proportion of women perceive the risks of side effects as more probable and as more dangerous than the risks of breast cancer [33, 34] and this is reflected in their becoming highly averse to the suggestion that a preventive therapy may produce a side effect [35]. In this background, it is also important to note that some studies [22, 33, 36] show a reasonable rate of women’s acceptance of preventive therapy, and therefore, it is possible to improve uptake.

Other barriers to preventive therapy [37] include poor commercial interest in the use of approved study medications due to concerns about influencing their established use [38] and liability issues, especially for off label use [39] and these will need to be tackled through changes in policy provisions.

## Strategies to improve uptake

Strategies to improve uptake of currently available preventive therapy start with increasing physician and patient awareness and countering prejudices. Refining risk prediction and patient communication would also be valuable. Research needs and strategies to move the cancer prevention field forward are discussed later in this paper.

### Countering prejudices – the issue of lack of reduction in mortality

Preventive therapy trials of SERMs [1] have not yet shown a reduction in breast cancer-specific or all-cause mortality, and this has been one of the main arguments [28, 29] against their use. Those not in favour of adopting therapeutic cancer prevention [28, 29] often fail to appreciate two issues; the lack of power of SERM trials to show mortality benefit at current length of follow-up and the substantial impact of just reducing cancer incidence.

The majority of SERM trials have a long follow-up, yet it is still too early to evaluate its effect on breast cancer mortality since breast cancer incidence still exceeds mortality by more than 10-fold [40], and therefore, the number of events needed to see a mortality impact is still inadequate. Furthermore, although IBIS-I (503 invasive breast cancers but only 57 breast cancer deaths) reported a 16-year median follow-up [40], the median follow-up after cancer diagnosis is only 8 years, which is still inadequate to evaluate effect on mortality [41].

The oncology community often relies on endpoints such as quality of life and in-hospital stay to determine the best treatment options in the absence of a clear impact on survival or mortality. Breast cancer incidence is still increasing worldwide and preventive therapy can avoid this dreaded diagnosis in a large number of women. This not only has potential individual benefits like avoiding a negative impact on the quality of life and avoiding treatments with substantial morbidity, but will also have a positive impact on healthcare systems in terms of improved allocation of limited treatment resources and thereby improving outcomes at a lower cost.

A somewhat similar issue has affected prostate cancer prevention by the use of 5α-reductase inhibitors (5-ARIs) as well [42]. A significant reduction in Gleason 6 or below cancers, and an increase in high-grade tumours was seen in prostate cancer prevention trial (PCPT) [43] and Reduction by Dutasteride of Prostate Cancer Events (REDUCE) trials [44], but not in observational studies [45]. A similar long-term survival in PCPT arms [46] and the evidence for potential cost-effectiveness [47] of prevention by the use of 5-ARIs favour this intervention to reduce the ever increasing burden of prostate cancer.

### Increasing physician and patient awareness

Increasing physician and particularly patient awareness is essential. For example, media coverage that led to increased awareness of aspirin’s cancer prevention role [48] resulted in a more than 10-fold increase in its sales in the short term [49]. While this may be largely due to aspirin being a commonly used drug available over the counter, awareness does seem to play a role.

### Policy matters

Debates in medical journals often exclude the practical barriers to the implementation of preventive therapy. These need to be addressed at policy level, and therefore, engagement with policymakers is essential to eliminate such practical barriers. For example, poor commercial appeal of approved study medications [38] results in lack of licensing for preventive indications which in turn creates off-label use indemnity issues. Changes in policy could be brought about by new legislation and although yet unsuccessful, bills like the Off-patent Drugs Bill 2014–2015 presented in the UK parliament are a way forward [50].

## Research priorities to improve therapeutic cancer prevention

### Improving risk prediction – lessons from cardiovascular prevention

Unlike therapeutic cancer prevention, preventive therapy is very widely accepted and routinely used in the field of cardiovascular diseases. It is crucial to understand the lessons learned from cardiovascular medicine and also important to identify the differences so that alternative strategies or further research can be pursued for improving cancer prevention.

**Figure 2.**
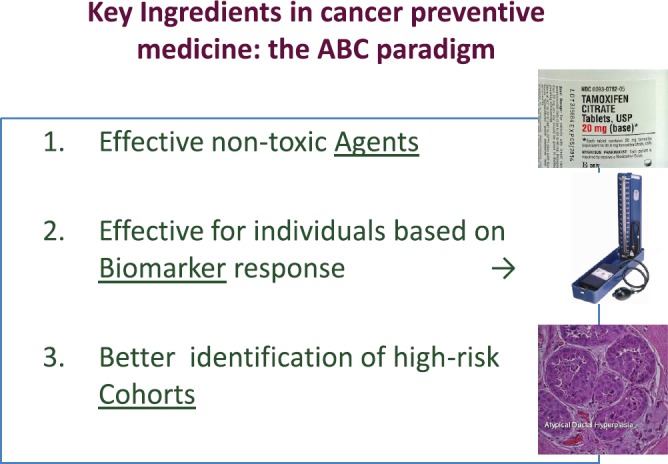
Key elements for cancer prevention: the ABC paradigm. (From De Censi A, Discussion abstract LBA504, ASCO June 5, 2011 [51].)

The key ingredients for the successful progress of cancer prevention are summarised in the ABC (agents–biomarkers, cohorts) paradigm [51] (Figure 2). However, the development of new agents in cancer prevention is hampered by the lack of suitable biomarkers and targets. Establishing breast cancer chemoprevention as standard clinical practice as has been done in cardiovascular medicine will require advances in many different fields, including biomarker research, the development of more powerful tools to identify at risk individuals (cohorts), methods to better communicate the risks and benefits of treatments, and establishing innovative trial designs.

Risk factors such as high low-density lipoprotein (LDL) cholesterol or high blood pressure have been elevated to the status of a disease in cardiovascular prevention and health authority endorsement for treating these has been obtained. This has been possible not only because these risk factors correlate well with cardiovascular disease but also because these are good surrogates of response and can be frequently monitored by non-invasive means. Barring notable exceptions of a few genetic tests like breast cancer (BRCA) mutation, which serve as strong risk factors but are applicable to a very small fraction of the general population, and surrogates like change in breast density for tamoxifen response [52], there is a great dearth of risk factors and surrogate markers of response in the field of therapeutic cancer prevention.

Two important types of risk factors play a role from the preventive therapy perspective: risk factors for the disease in question and factors for predicting the risk of side effects of a particular preventive therapy. The relative importance of these is influenced by the spectrum of cancers that could be prevented. For example, aspirin has preventive effects in a range of different cancers [48, 53], so that identification of high-risk individuals is not only difficult, but also not so necessary. On the other hand, aspirin’s side effects are severe only in a small proportion of individuals [54] so that the ability to reliably identify such individuals and not offer aspirin to them becomes more important for improving the overall benefit-harm profile of the drug for cancer prevention. In contrast, the benefits of endocrine agents are expected only for breast cancer, and a key priority is to better identify women at high risk of developing this specific disease.

Refining existing biomarkers and development of new biomarkers of cancer risk, response to therapy, and the risk of side effects of agents is urgently needed. Recent developments in the field of cell-free DNA (cfDNA)/circulating tumour DNA (ctDNA) [55] may provide an opportunity identify/develop biomarkers of cancer risk and response to therapy that can be repeatedly monitored in a non-invasive manner. Assessment and validation of newer individualised and preference-sensitive interactive tools is also needed to improve patient communication [56].

### Revisiting existing agents and drug re-purposing

Improving and individualising our estimation of benefit–harm balance alone will, however, not suffice as the findings from behavioural research repeatedly suggest that the perception of side effects is one of the main barriers. It is therefore also essential that drugs used for cancer prevention not only have high efficacy but also have a very safe side effect profile from an at-risk individual’s perspective. New compounds may not be able to overcome this perception hurdle, but, repurposed drugs that are widely used for other purposes (e.g. aspirin, metformin) may be more acceptable as evidenced by the aspirin example above [49]. Similarly, dosing of existing agents could also be altered to improve the side effect profile without losing efficacy.

One simple strategy is to lower the dose of drugs that are in common use in the adjuvant setting based on the notion that prevention of cancer cells could occur at a lower dose than eradicating established tumour cells. This was done for antihypertensive agents in the polypill for stroke prevention in the general population [57]. A second approach is to adopt an intermittent administration similarly to that used in the chemotherapy setting in an attempt to minimise risks while retaining benefits. A meta-analysis of the adjuvant tamoxifen clinical trials reported that the benefits of tamoxifen in terms both of recurrence and mortality appeared to be equivalent between 20 mg/day and 30–40 mg/day [58]. In a randomised biomarker trial conducted in the late 1990s [59], a dose reduction to 10 mg every other day did not affect the activity of tamoxifen on total cholesterol, insulin-like growth factor 1 (IGF-1) and C-reactive protein using an ultrasensitive assay. In a window of opportunity three-arm randomised trial, the antiproliferative effects of tamoxifen at 1 mg/day and 5 mg/day relative to those of the standard dose of 20 mg/day were assessed on Ki-67 labelling index (LI) in breast tumour specimens using a pre-surgical model in 120 breast cancer patients [60]. Labelling of Ki-67 decreased in all three tamoxifen dose-level groups, with no difference in the magnitude of reduction among the groups (*p* = 0.81). In the hormone replacement therapy (HRT) opposed by low-dose tamoxifen (HOT study), a multicentre phase-III trial in 1884 postmenopausal healthy women, current or *de novo* HRT users were randomised to either tamoxifen 5 mg/day or placebo for 5 years, with 5 years of follow-up. A trend towards a beneficial effect from low-dose tamoxifen in reducing BC events was observed (risk ratio (RR): 0.80; 95% CI: 0.44–1.46) with greater efficacy being seen in luminal-A tumours (RR: 0.32; 95% CI: 0.12–0.86), HRT users <5 years (RR: 0.35; 95% CI: 0.15–0.82) and women completing at least 12 months of treatment (RR: 0.49; 95% CI: 0.23–1.02) [61]. The utility of low-dose tamoxifen as preventive therapy is also being evaluated in a different cohort of high-risk women such as women with oestrogen receptor-positive ductal carcinoma *in situ* (ER-positive DCIS) [62, 63] and Hodgkin’s lymphoma survivors who were previously treated with ≥ 15 Gy of mantle irradiation to the chest before age 30 [64].

Similarly, aspirin’s preventive benefits on cancer have been seen for all daily doses above 75 mg and, in the absence of greater efficacy at higher doses, low-dose aspirin appears to be a reasonable choice for cancer prevention in view of a lower risk of side effects [48, 54].

Along this line, additional agents may be repurposed as preventive medications after careful determination of the minimal effective dose. For example, exemestane, an irreversible aromatase inhibitor, has showed an overall 65% reduction in all breast cancers and a 73% reduction specifically for ER-positive breast cancer in the intervention arm compared to the placebo group [3]. However, the standard dose employed in the treatment setting was used also for prevention, and the benefit-harm ratio of adverse effects such as bone density loss, musculoskeletal pain, and climacteric syndrome associated with treatment may be less favourable in the prevention setting [65]. Since the oestradiol suppression persists for at least 5 days after administration of a single dose [66], it seems reasonable to consider reducing the dose or changing the administration schedule, while still maintaining a significant oestradiol reduction. A dose of 10 mg daily of exemestane has been shown to be equivalent to 25 mg for suppressing oestradiol levels [67]. A window of opportunity biomarker trial has been planned in which participants will be randomised to receive either exemestane 25 mg/day or 25 mg/ three times a week or a single dose of 25 mg/week for 5 (±1) weeks. The primary endpoint will be the change in serum oestradiol using an ultrasensitive assay after 5 (±1) weeks treatment [68].

### Strategic shift to more preventive research

The failure [69] of agents like beta carotene [70–73] and vitamin E [74–76] in large trials of lung cancer prevention underscores the need for identification of newer targets and agents, which can only be accomplished through increased preventive research. The pharmaceutical industry’s investment in the development of cancer preventive agents is disproportionately low as compared to that in the development of agents for cancer treatment. Industry’s focus on profits means that this imbalance is unlikely to be corrected in the foreseeable future and therefore academia and other non-pharmaceutical institutions, including government agencies need to take a more active role in the development of agents for cancer prevention. The development of a new drug requires substantial investment which is not readily available through academic research funding, whereas repurposing of a drug for new indication is likely to cost substantially less. Drug repurposing could provide a more achievable focus for developing therapeutic cancer prevention and is one in which academia can play a major role.

## Conclusions

Cancer incidence continues to increase worldwide and our war on cancer cannot be won without effective cancer prevention measures that include lifestyle modifications as well as therapeutic prevention. Despite the availability of several efficacious cancer prevention agents, uptake of cancer preventive therapy remains woefully inadequate. Increasing the awareness of preventive therapy among physicians – along with highlighting the important differences between preventive therapy and cancer treatment so as to eliminate prejudices against preventive therapy is necessary. Improving cancer risk and drug response prediction to maximise the benefit–harm balance, coupled with repurposing and improving the safety profile of existing agents by experimenting with dose is urgently needed. The development of newer preventive agents will not be enough by itself and engagement with policymakers cannot be ignored in order to remove practical obstacles to implementation of existing effective agents.

## Conflicts of interest

The authors declare that there are no conflicts of interest.

## Funding

This study was partially supported by Gruppo Bancario Credito Valtellinese, and Cancer Research UK programme award (C569/A16891).

Smith is supported by a Cancer Research UK Postdoctoral Fellowship (C42785/A17965).

## Disclaimer

The findings and conclusions in this report are those of the authors and do not represent the official position of the authors’ respective institutions.

## Figures and Tables

**Table 1.** Barriers to preventive therapy and strategies to overcome these barriers.

Barriers	Strategies to overcome barriers
Physicians’ lack of knowledge/prejudices.	Increasing physician awareness and countering prejudices.
Individual’s lack of knowledge.	Improving physician–patient communication and information sharing; educational interventions.
Individual’s fear of side effects.	Exploring re-purposing of commonly used agents with well-documented safety profile.
Underestimation of benefits and/or overestimation of harms.	Acknowledging different needs of risk prediction for different diseases and agents. Refining risk prediction and risk communication. Development of biomarkers that can be frequently monitored by non-invasive means.
Adverse effects of agents.	Exploring strategies to reduce adverse effects, e.g., dosing modifications.
Lack of well-proven agents for several cancers.	Increased focus on preventive research, particularly in academia.
Licensing and off-label use issues.	Policy engagement.
